# Reconciling nature conservation and traditional farming practices: a spatially explicit framework to assess the extent of High Nature Value farmlands in the European countryside

**DOI:** 10.1002/ece3.1415

**Published:** 2015-02-05

**Authors:** Angela Lomba, Paulo Alves, Rob H G Jongman, David I McCracken

**Affiliations:** 1 Centro de Investigação em Biodiversidade e Recursos Genéticos (CIBIO), Campus Agrário de Vairão4485-661, Vairão, Portugal; 2 Alterra, Wageningen University and Research CentreP.O. Box 47, 6700 AA, Wageningen, the Netherlands; 3 Hill & Mountain Research Centre, Scotland's Rural CollegeAuchincruive, Ayr, KA6 5HW, UK

**Keywords:** Agri-environment schemes, agro-biodiversity, conservation and monitoring programs, indicators, low-intensity farming practices

## Abstract

1. Agriculture constitutes a dominant land cover worldwide, and rural landscapes under extensive farming practices acknowledged due to high biodiversity levels. The High Nature Value farmland (HNVf) concept has been highlighted in the EU environmental and rural policies due to their inherent potential to help characterize and direct financial support to European landscapes where high nature and/or conservation value is dependent on the continuation of specific low-intensity farming systems.
2. Assessing the extent of HNV farmland by necessity relies on the availability of both ecological and farming systems' data, and difficulties associated with making such assessments have been widely described across Europe. A spatially explicit framework of data collection, building out from local administrative units, has recently been suggested as a means of addressing such difficulties.
3. This manuscript tests the relevance of the proposed approach, describes the spatially explicit framework in a case study area in northern Portugal, and discusses the potential of the approach to help better inform the implementation of conservation and rural development policies.
4. Synthesis and applications: The potential of a novel approach (combining land use/cover, farming and environmental data) to provide more accurate and efficient mapping and monitoring of HNV farmlands is tested at the local level in northern Portugal. The approach is considered to constitute a step forward toward a more precise targeting of landscapes for agri-environment schemes, as it allowed a more accurate discrimination of areas within the case study landscape that have a higher value for nature conservation.

## Introduction

Over past centuries, European landscapes have been shaped by human management. Traditional, low-intensity agricultural practices, adapted to local climatic, geographic, and environmental conditions, led to a rich, diverse cultural and natural heritage, reflected in a wide range of rural landscapes, most of which were preserved until the advent of industrialized agriculture (Bignal & McCracken 2000; Paracchini et al. 2010; Oppermann et al. 2012).

Agricultural landscapes currently account for half of Europe's territory (Overmars et al. 2013), with ca. 50% of all species relying on agricultural habitats at least to some extent (Kristensen 2003; Moreira et al. 2005; Halada et al. 2011). Due to their acknowledged role in the maintenance of high levels of biodiversity, low-intensity farming systems have been highlighted as critical to nature conservation and protection of the rural environment (Beaufoy et al. 1994; Paracchini et al. 2010; Halada et al. 2011; Egan & Mortensen 2012). Many areas included in the Natura 2000 network, the main policy initiative for nature conservation in the European Union, are currently under agricultural management for crop or livestock production. Maintaining such High Nature Value farming systems is crucial for the long-term success of Natura 2000 as a fundamental ecological network in Europe (EEA 2004).

The concept of “High Nature Value farmlands” (hereafter HNVf; Beaufoy et al. 1994) was devised to help characterize and direct financial support to those agriculture-dominated landscapes where high nature and/ or conservation value is dependent on the continuation of specific low-intensity farming systems (Andersen et al. 2003; Pedroli et al. 2007; Halada et al. 2011; Ribeiro et al. 2014). HNVf owe their intrinsic ecological value to the presence of semi-natural agricultural habitats (defined after Andersen et al. 2003 as type 1, hereafter HNVf_1_), to the presence of small crop fields intermingled with other farmland features such as mature trees, shrubs, scrub, or linear features such as field margins and hedges (defined after Andersen et al. 2003 as type 2, hereafter HNVf_2_), and to the presence of species of high conservation interest (e.g., bird, reptiles), in often intensively managed landscapes (defined after Andersen et al. 2003 as type 3, hereafter HNVf_3_).

While farmlands of high nature value and their associated management practices have been widely acknowledged as beneficial for biodiversity enhancement (e.g., Bignal & McCracken 2000; Egan & Mortensen 2012), such landscapes have been declining due to rural depopulation, agricultural abandonment and afforestation in marginal farming areas, and intensification in the most productive areas (Stoate et al. 2009; EEA 2012; Oppermann et al. 2012; Ribeiro et al. 2014). As a consequence, the importance of HNVf for nature conservation and rural development is now enshrined within Europe's agricultural and environmental policies (Stoate et al. 2009; Jongman 2013; Ribeiro et al. 2014), and assessing changes to the area of agricultural land under HNVf is currently one of the biodiversity indicators used to evaluate the effectiveness of EU Member State Rural Development Programs (RDPs; EC 2006; Peppiette 2011).

Assessing the extent of HNV farmland by necessity relies on both ecological and farming systems' data, and difficulties with making such assessments have been widely described (Peppiette 2011; Oppermann et al. 2012; Lomba et al. 2014). While EU common methodological guidelines broadly rely on land cover, farming system and species data to identify HNV farmlands extent, condition, and dynamics (Andersen et al. 2003; EC 2006; Paracchini et al. 2008; EENRD 2009; Peppiette 2011; Lomba et al. 2014), the diversity of rural landscapes across the EU, the lack of suitable datasets on essential indicators, and especially the absence of a common methodology for mapping currently constrain the operationalization of the HNVf concept as a policy instrument across Europe (Pedroli et al. 2007; Peppiette 2011; EEA 2012; Oppermann et al. 2012). Hence, the identification, testing, and implementation of effective spatially explicit indicators that could be used to express landscape and/or crop heterogeneity in relation to known biodiversity levels and management practices have been encouraged (Wascher et al. 2010; EEA 2012; Lomba et al. 2014).

In this manuscript, a spatially explicit framework is presented after Lomba et al. (2014) to assess the extent of HNVf at the local administrative unit level (LAU, as defined by Eurostat; http://epp.eurostat.ec.europa.eu/). Lomba et al. (2014) advocate that a common European framework for the identification, mapping, and regular assessment (i.e., monitoring) of HNVf areas should rely on the highest spatial and temporal resolution data available within each administrative unit and implemented in each targeted area, ensuring that the most accurate data are always mobilized to help identify HNVf and assess rural development programs at a local, national, and EU level (RDPs; EC 2006; but see Lomba et al. 2014 for a review). Overall, our HNVf mapping approach relies on the spatially explicit analysis and combination of sets of indicators known to express the most relevant ecological and management features of agro-ecosystems (Lomba et al. 2014), that is, data expressing landscape structure and composition (Landscape Elements indicators), farming systems (Extensive Practices indicators), and crop diversity (Crop Diversity indicators). Additionally, information on species whose survival is dependent on the maintenance of extensive farmlands is also included (Indicator Species). The proposed approach is illustrated for a municipality located in a mountainous area of northern Portugal. The proposed spatially explicit approach and its outcomes in the study area are discussed in the context of land-sharing for biodiversity conservation and/ or enhancement in the EU countryside, together with its potential application to HNVf assessment across Europe and to helping improve the targeting of agri-environment schemes.

## Materials and Methods

### Study area

**Figure 1:**
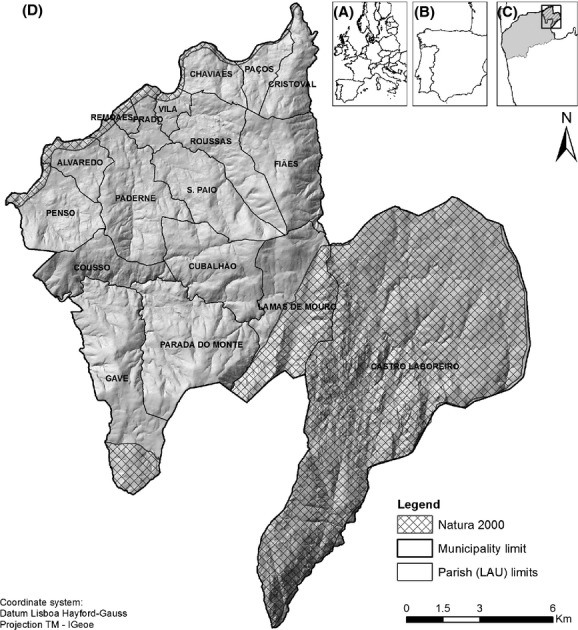
The study area, Melgaço municipality and encompassed parishes (D), and its geographic location in the European (A), Iberian (B), and Portuguese territories (C).

The municipality of Melgaço, located in a mountainous area of northern Portugal (Minho-Lima region, NUTS III; Eurostat, http://epp.eurostat.ec.europa.eu/) between latitudes of 41°55′20″ and 42°9′11″N and longitudes of 8°4′52′ and 8°20′32″W (Fig.1), includes 18 civil parishes, each of which corresponds to a local administrative unit (LAU 1). The whole area is considered a Less Favoured Areas (LFA) is a EU legal designation, so it is not supposed to be changed across text. and, more specifically, classified as a mountain/hill area according to the article 3.3 of Directive 75/268/EEC (e.g., Beilin et al. 2014). The southeastern part of Melgaço is part of the Peneda-Gerês National Park (Fig.1), a protected area with ca. 70,000 ha, classified also as Site of Community Importance (SCI, PTCON0001) and Special Protection Area (SPA; PTZPE0002) of the EU Natura 2000 network. The northern part is included in the fluvial SCI “Rio Minho” (PTCON0019).

Melgaço's landscapes include a mixture of lowland areas, large valleys, and mountain massifs. Being a LFA, Melgaço's natural handicaps (mostly related to altitude, steep slopes, poor soils, harsh climatic conditions, and isolation) shaped the agricultural landscapes, which are characterized by a pattern of small and fragmented low-intensity traditional farms, which produce mainly for self-consumption (Pôças et al. 2011; Lomba et al. 2012; Beilin et al. 2014). Such traditional agro-pastoral systems have shaped two types of landscape mosaics: (1) open grazing lands (“outfields'', mainly “baldios”) with oligotrophic soils, dominated by heath, low scrub and mesic, acidophilous grasslands at plateau and summit areas and (2) forest-rich agricultural lands (“infields'') on nutrient-rich soils at the bottom of slopes and valleys, where hay meadows are the dominant elements, and where forest patches are managed for wood and water regulation services (Aguiar et al. 2010; Cerqueira et al. [Bibr b10],[Bibr b505]; Lomba et al. 2012). These traditional agro-ecosystems not only include agricultural areas but also incorporate vast mountain areas which provide important natural pasture lands and sources of bedding for animals as well as firewood (Pôças et al. 2011; Maxted 2012). In lowland areas with a Mediterranean climate, farmland is usually located in mild slopes around rural villages and includes important areas of vineyards, as well as cereal fields and other annual crops. The steepest slopes are occupied by forest stands planted with *Pinus pinaster* Aiton and *Eucalyptus globulus* Labill. subsp. *globulus*. Overall, dominant HNVf types include the high-altitude irrigated pastures (also known as “lameiros”), small terraces, and extensive communal grazings (“baldios”) as HNVf_1_ (Oppermann et al. 2012), and the highly diverse complex mosaics of arable and horticultural crops, with vineyard and orchards, where small-scale livestock graze permanent pastures, often intermingled with arable land, as HNVf_2_ (Moreira et al. 2005; Oppermann et al. 2012). Due to their characteristic biophysical constraints, traditional mountain farming systems, such as those observed in Melgaço, are facing collapse as a consequence of agricultural abandonment (Lomba et al. 2012; Beilin et al. 2014).

### Spatially explicit framework and proposed indicators to assess HNVf extent

**Figure 2:**
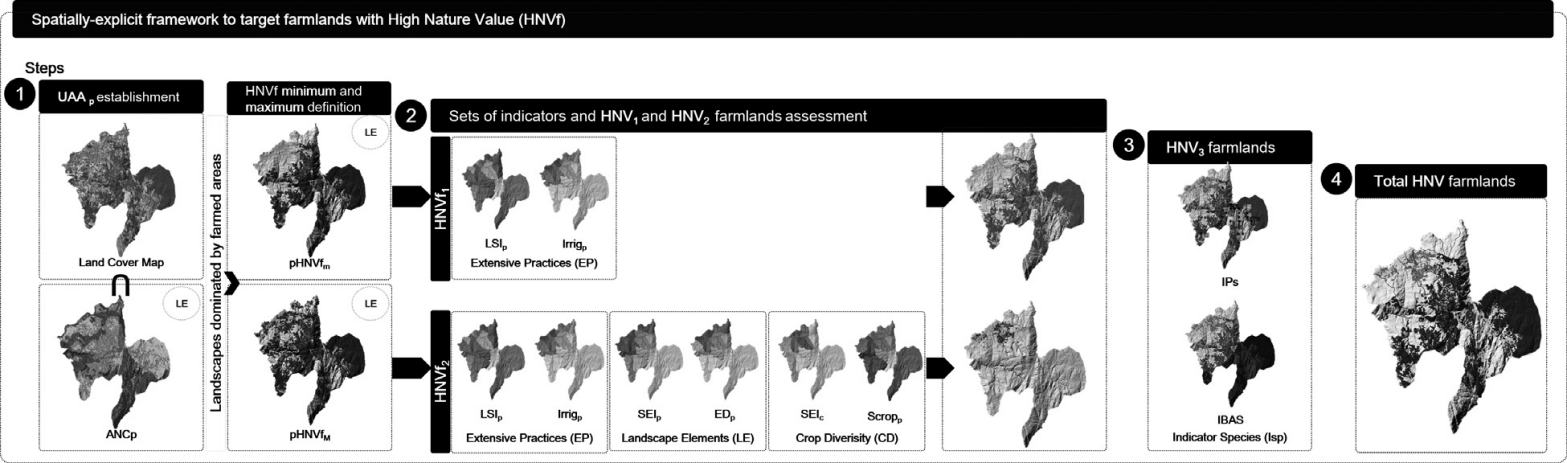
Spatially explicit approach to assess High Nature Value farmlands (HNVf types 1, 2, and 3). In Step 1, indicators reflecting landscape composition (LE) were applied to ascertain the utilized agricultural area (UAA), the dominance of agriculture at the landscape level (parish), and areas with high or moderate potential to be HNVf, assumed to be suitable to target HNVf_1_ and HNVf_2_, respectively. In Step 2, indicators expressing the intensity of Farming Practices (EP) were applied to discriminate parishes that contain HNVf1; the intensity of Farming Practices, Landscape Elements, and Crop Diversity information (CD) were applied to identify HNVf_2_. The need to identify any additional areas of HNVf_3_ was determined in Step 3, using information regarding Indicator Species. The total extent of HNVf was assembled in Step 4.

The backbone of the framework, outlined in Fig.2, is the spatially explicit assemblage of distinct sets of information acknowledged as relevant data for HNV farmland assessment (Beaufoy 2008; Lomba et al. 2014). Although challenging, effective identification of agriculture-dominated areas, their degree of naturalness, and the underlying farming practices are essential for common HNVf mapping and monitoring across EU rural landscapes. Four sets of indicators are proposed: (1) landscape elements; (2) extensive practices; (3) crop diversity; and (4) indicator species (cf. Fig.2 and Table1; see Lomba et al. 2014), to provide information on landscape structure and composition, intensity and diversity of agricultural practices and on the occurrence of species of nature conservation value, respectively. Table1 provides a detailed description of each set of indicators, the underlying rationale for their selection, and the type of HNVf which these assess. These indicators are built on the common EU guidelines for the HNVf indicator implementation and aim to express proxies regarding land use, crop diversity, and farming systems (EENRD 2009). We advocate that such framework can support HNVf mapping and monitoring across EU countryside, as it is flexible enough to allow the mobilization of the best spatial and temporal resolution data within each targeted administrative unit (in each MS), while still complying with a common set of indicators.

**Table 1:** Indicators used to implement the spatially explicit approach to assess the extent of High Nature Value farmlands. Indicators expressing landscape characteristics (Landscape Elements), the intensity (Extensive Practices), and the diversity (Crop Diversity) of farming practices, as well as occurrence of Indicator Species, are described and the rationale underlying their selection presented. %, stands for the percentage; n.a., nonapplicable

Designation	Code(s)	Description and rationale	Source and Resolution	HNVf type	Reference
**Landscape Elements (LE)**					
Natural constraints for agriculture	ANC_p_	Areas with natural constraints to agricultural per parish; for details see Supplementary Information S2, used to define areas suitable for agricultural practices.	n.a.	HNVf type 1	Van Orshoven et al. (2012)
				HNVf type 2	
Farmlands dominance in the landscape	P.UAA_p_	Areas with higher percentages (%) of farmlands UAA (P.UAA_p_) when comparing to areas covered by forests (P.Forest_p_).	Associação de Municípios do Vale do Minho (2009)	HNVf type 1	Beaufoy et al. (1994), Andersen et al. (2003)
	P.Forest_p_			HNVf type 2	
				HNVf type 3	
Minimum–Maximum HNV farmland areas	pHNVf_m_	Farmlands more likely to represent HNVf (Minimum, pHNVf_m_) in the study area (Melgaço municipality) and *per* parish (Supporting Information S1)		HNVf type 1	Andersen et al. (2003), Paracchini et al. (2006)
	pHNVf_M_	Farmlands with moderate potential to represent HNVf (Maximum, pHNVf_M_) in the study area (Supporting Information S1)		HNVf type 2	
Shannon's Diversity Index	SDI_p_	More diverse landscapes, with land cover types evenly distributed, are expected to have higher biodiversity.	Associação de Municípios do Vale do Minho (2009)	HNVf type 2	Tscharntke et al. (2005), BioBio (2012)
Shannon's Evenness Index	SEI_p_				
Patch number	NP_p_	Number of patches is often used as an indicator for landscape fragmentation, which sometimes is considered beneficial for agro-biodiversity.			Aavik & Liira (2009), Armengot et al. (2011)
Mean shape index	MSI_p_	Higher MSI values occur in natural and semi-natural landscapes.			Tscharntke et al. (2005)
Edge density	ED_p_	Density of edges (m/ha; meters per hectare) in relation to the parish area is relevant to wildlife maintenance as they constitute semi-natural habitats.			BioBio (2012)
**Extensive Practices (EP)**					
Livestock density index	LSI_p_	The number of livestock units per hectare of the UAA (LSU_i_/ha of UAA) per parish. Lower values of LSU_i_ highlight the dominance of semi-natural forage, including grasslands and often scrub, woodlands, or a combination of several types.	INE (2009)	HNVf type 1	Pointereau et al. (2007), EENRD (2009), BioBio (2012)
				HNVf type 2	
Share of irrigated area	Irrig_p_	Share of irrigated area per total of UAA in each parish. Proxy for agricultural intensification.			Pointereau et al. (2007)
**Crop Diversity (CD)**					
Shannon's Evenness Index for Crop Diversity	SEI_c_	Crop diversity, associated with low inputs, and a network of natural and/or semi-natural features, express a high nature value for biodiversity conservation in farmlands.	INE (2009)	HNVf type 2	Andersen et al. (2003), Paracchini et al. (2008), BioBio (2012)
Crop richness	SCrop_p_	Number of crop types cultivated per parish. Lower specialization contributes to higher levels of biodiversity in agricultural landscapes.		HNVf type 2	BioBio (2012)
**Indicator Species (Isp)**					
Important Bird and Biodiversity Areas	IBAs	IBAs are sites needed to ensure the survival of viable populations of most world's bird species.	1:20,000	HNVf type 3	Andersen et al. (2003), Paracchini et al. (2008)
			SPEA (2003)		
Indicator plant species	IPs	Plant species under conservation status (Habitats Directive) depending on extensive farmlands maintenance can provide additional information for the identification of HNVf areas (*Angelica laevis, Paradisea lusitanica*,*Senecio doria legionensis*, and *Veronica micrantha*).	Personal database		

The utilized agricultural area (UAA) was ascertained from a fine-scale land cover map (Step 1, see Table S1, Appendix S1 in Supporting Information for details). Here, individual landscapes were taken to be each one of the individual parishes that constitute the municipality. Classes expressing farmed areas and land cover classes covering areas off the farm (e.g., grazed heathlands and other grazing areas in common usage), known to express other semi-natural areas used as forage of fodder resources, were selected, and the total UAA per parish was determined (IEEP 2010; Oppermann et al. 2012). Data reflecting natural constraints for agriculture (ANC_p_; as defined by Van Orshoven et al. 2012; for detailed information see Table S2; Appendix S2 in Supporting Information) were applied, so that only heathlands under no or moderate limitations to agriculture were included. This enabled the identification of off-farm grazing areas, which are known to constitute a large proportion of HNVf in some regions (IEEP 2010; EEA 2012). Dominance of farmlands was established on the basis of the share of agriculture (P.UAA_p_; Table1) and forest (broad-leaved, coniferous and mixed forests identified in the land cover map; P.Forest_p_; Table1) per parish. For the eligible parishes, land cover classes associated with agricultural practices (i.e., coincident with the established UAA) were classified according to their potential to exhibit high nature value following the minimum–maximum approach (Andersen et al. 2003; Paracchini et al. 2008; IEEP 2010; for detailed information see Appendix S1). As a result, the spatial representation of putative “extremes” within which HNVf was likely to occur was obtained and used as component of the “Landscape Elements” set. The outcomes from such approach correspond to areas with very high likelihood (corresponding to land cover classes known to consist primarily of HNVf; *minimum* HNVf areas; pHNVf_m_, Fig.2) and moderate likelihood (including other potential HNVf classes, depending on the farming intensity; *maximum* HNVf areas; pHNVf_M_, Fig.2) of being HNVf_1_ and HNVf_2_ farmlands, respectively.

As land cover maps do not convey information on the land use intensity (Lomba et al. 2014), in Step 2 of the proposed framework additional information expressing the prevalence of a high proportion of semi-natural vegetation, the diversity of elements at the landscape level (Landscape Elements indicators), the extensive character of the farming practices (Extensive Practices indicators), and the diversity of crops (Crop Diversity indicators) were applied to refine the identification of HNVf_1_ and HNVf_2_. In Step 3, Indicator Species were used to asses areas of HNVf_3_. Table1 presents the indicators included in each indicator set, a short description and the underlying rationale, the units and scale and/or resolution (when applicable), and relevant supporting references (for full information see Appendix S3 in Supporting Information).

A more refined HNVf_1_ assessment was achieved by overlaying the *minimum* HNVf areas and, sequentially, the livestock density index (LSI_p_) and the share of irrigation (Irrig_p_; Fig.2 and Table1). This allowed the identification of landscape parishes under more extensive agricultural practices. To refine assessment of HNV farmlands of type 2, the *maximum* HNVf map (which included other farmlands with potential to be of HNV, for example, mosaics of arable land and grasslands; cf. Appendix S1), and the three sets of indicators were combined. Farmlands under more intensive agricultural practices, expressed as higher values of LSI_p_ and Irrig_p_, were considered and overlaid with indicators expressing small-scale features of the landscape and the diversity of crops (Landscape Elements and Crop Diversity indicators, respectively; cf. Fig2 and Table1). Step 2 resulted in the spatial identification of HNV types 1 and 2 in the study area.

To test the sensitivity of our approach and to identify any potential HNVf_3_ areas, data on the distribution of four plant species of recognized conservation value and dependent on agricultural-related habitats (Step 3, Fig.2 and Table1; Paracchini et al. 2008) were compared against HNVf_1_ and HNVf_2_ maps, and their coincidence was analyzed. Information on indicator plant species was complemented with consideration of the location of Important Bird Areas (IBAs; Paracchini et al. 2008). Assessing the coincidence of the HNVf areas identified in Step 2 with the known occurrence of Indicator Species (Step 3) is essential to highlight any need to include additional complementary HNVf areas that otherwise would not be identified due to the intensity of the agricultural practices.

Spatial analysis was implemented in ArcGIS 10.2 for Desktop (ESRI 2009), and landscape metrics were calculated with Patch Analyst 5.1 (Rempel et al. 2012), considering that each landscape is coincident with individual parishes. Landscape metrics were computed considering all classes for each parish, and considering only farmland areas per parish, to assure that landscape patterns are able to express small-scale patterns. As both patterns were found to be similar, only metrics at the landscape level were considered to comply with HNVf operationalization requirements (Lomba et al. 2014) and thus retained for all analysis. Sets of indicators presented in Table1 were tested for correlation by Kendall's *τ* index (a nonparametric index suitable for low number of cases), and a value of 0.7 established as a maximum threshold for indicators was considered. Overall, threshold values for the indicators applied (HNVf_1_ and HNVf_2_) were selected as being those enabling a more clear segregation between parishes, and groups' robustness was tested with cluster analysis techniques (Statsoft, 2013).

## Results

**Figure 3:**
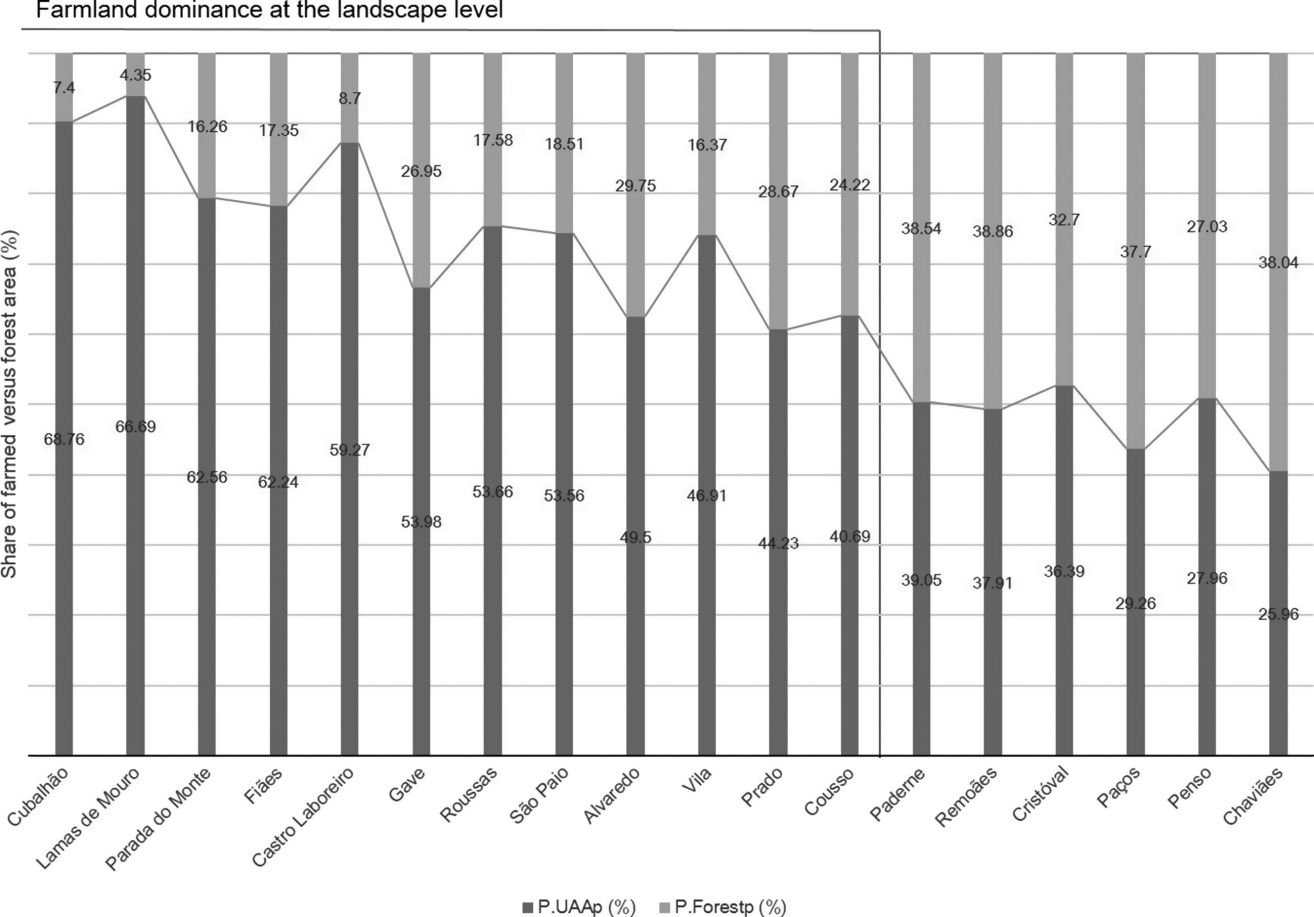
Relationship between shares of farmland (P.UAA_p_) *versus* forest (P.Forest._p_) areas for each parish within Melgaço municipality. Areas are expressed as hectares (ha). Share of farmlands (P.UAA_p_) and forests (P.Forest_p_) is presented as percentage (%) of the respective cover in relation to the parish area (*T*_area_). *n.f*. stands for not farmland areas. Gray line highlights the threshold considered.

The spatially explicit expression of the share of farmed (P.UAA_p_) and forested (P.Forest_p_) areas in each of the 18 Melgaço's parishes is represented in Fig.3 (for detailed information see Table S4.1 on Supporting Information Appendix S4).

Overall, the values of UAA per parish ranged from 25.96% in Chaviães to 68.76% in Cubalhão (cf. Fig.3). Conversely, the lowest value of forested areas was in Lamas de Mouro (4.30%) and the highest in Remoães (38.9%). The analysis of farmed versus forested areas, presented in Fig.3, highlighted the farmland dominance at the landscape level (i.e., parish level) for 12 of the 18 parishes. As a rule of thumb, a percentage of 40% was established to define the dominance of farmed areas in the landscape, thus excluding Chaviães, Cristóval, Paços, Paderne, Penso e Remoães, as legible parishes for HNVf assessment.

### Landscape and farming system indicators for the assessment of HNVf_1_ and HNVf_2_

**Figure 4:**
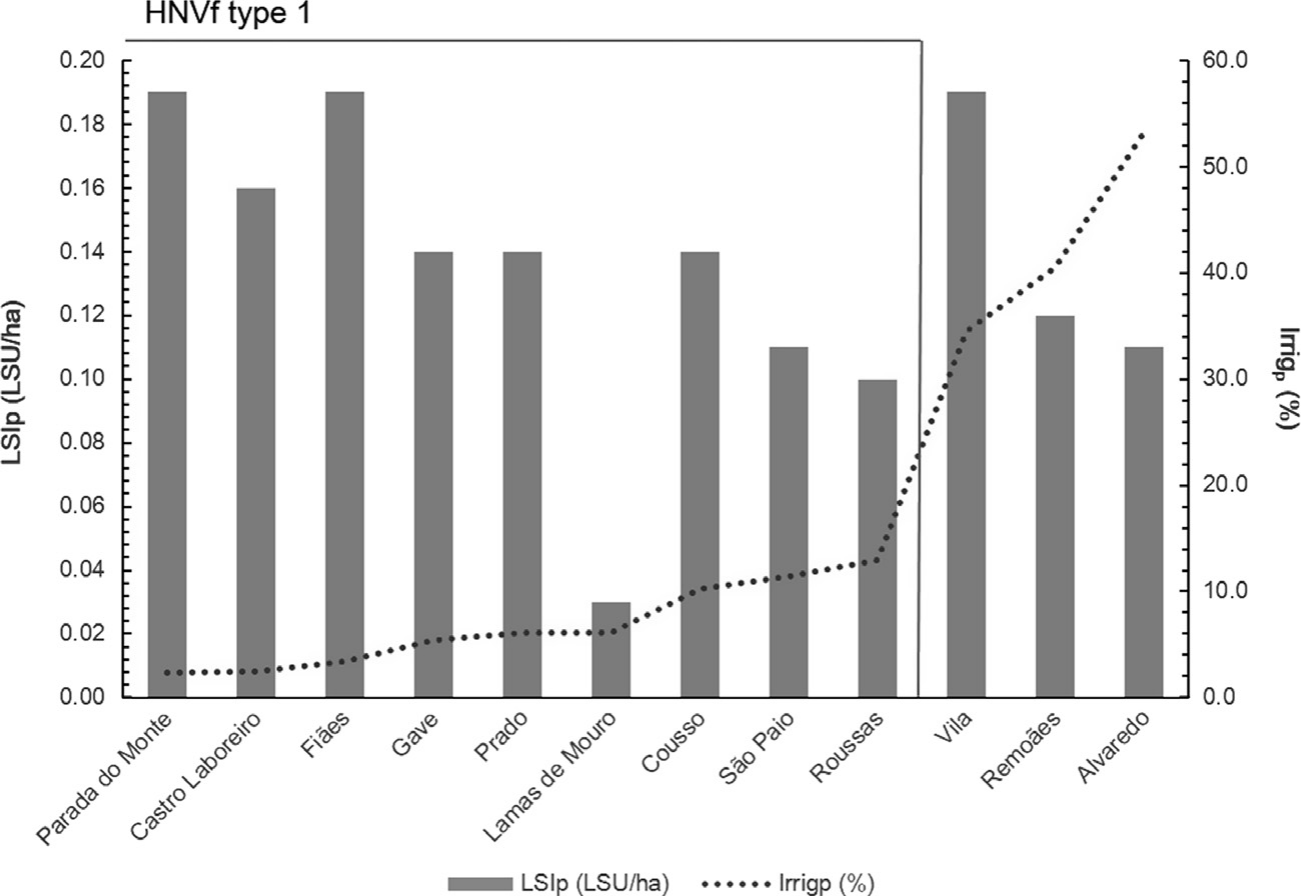
Rank of parishes according to extensive practices indicators, livestock density index (LSI_p_), and share of irrigated area (Irrig_p_). Gray line highlights the threshold considered to assess High Nature Value farmland (HNVf) type 1 extent.

**Figure 5:**
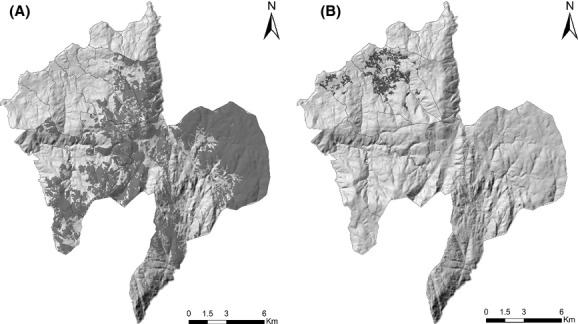
Areas identified as High Nature Value farmlands type 1 (A) and type 2 (B) in the study area. Black lines represent the geographic boundaries of Melgaço's parishes.

Implementation of Step 2 (cf. Fig.2) resulted in the discrimination between HNVf_1_ and HNVf_2_ (Fig.4 and Table2, respectively; Fig.5). Figure4 (for full information see Appendix S4 in Supporting Information) shows the relation between values established as thresholds for the indicators of intensity of agricultural practices. Livestock density and the share of irrigated areas at the parish level were analyzed to assess HNVf_1_. As all values for LSI_p_ were found to be under 0.2 LSU/ha, values of Irrig_p_ above 15% of the total UAA were considered as a threshold for assessing HNVf_1_. As a result, the parishes Vila, Prado, and Alvaredo were excluded.

**Table 2:** Rank of parishes according to Extensive Practices, Landscape Elements, and Crop Diversity indicators, with gray area highlighting thresholds considered for each set of indicators to assess HNVf type 2 extent. All areas are expressed as hectares (ha)

Sets of Indicators	Extensive practices		Landscape elements										Crop diversity			
Parish	LSIp (LSU/ha)	Irrig_p_ (%)	Parish	SEI_p_	Parish	SDIp	Parish	NP_p_	Parish	MSI_p_	Parish	ED_p_ (m/ha)	Parish	SDIc	SEI_c_	SCrop_p_
Lamas de Mouro	0.03	2.38	Prado	0.86	Alvaredo	2.68	Gave	212.00	Vila	2.60	Vila	448.22	São Paio	1.03	0.23	5.00
Roussas	0.10	12.94	Alvaredo	0.79	Prado	2.63	Roussas	174.00	Prado	2.57	Alvaredo	429.37	VIla	0.78	0.22	5.00
Alvaredo	0.11	53.24	Cousso	0.78	Cousso	2.61	São Paio	142.00	Spaio	2.47	Prado	420.47	Roussas	0.75	0.17	5.00
São Paio	0.11	11.44	São Paio	0.75	Roussas	2.55	Fiães	137.00	Fiães	2.33	Roussas	338.17	Prado	0.72	0.18	5.00
Cousso	0.14	10.19	Roussas	0.74	São Paio	2.31	Alvaredo	126.00	Alvaredo	2.31	São Paio	318.64	Alvaredo	0.58	0.12	5.00
Gave	0.14	6.16	Vila	0.71	Gave	2.28	Cousso	111.00	Roussas	2.22	Cousso	272.49	Gave	0.51	0.10	7.00
Prado	0.14	40.50	Gave	0.69	Vila	2.13	Prado	59.00	Gave	2.09	Fiães	272.15	Cousso	0.32	0.05	7.00
Castro Laboreiro	0.16	2.50	Fiães	0.64	Fiães	2.01	Vila	42.00	Cousso	2.09	Gave	238.81	Fiães	0.11	0.02	4.00
Fiães	0.19	5.45	Parada do Monte	0.58	Parada do Monte	1.97										
Parada do Monte	0.19	6.14	Cubalhão	0.56	Castro Laboreiro	1.65										
Vila	0.19	34.44	Lamas de Mouro	0.48	Cubalhão	1.60										
Cubalhão	0.23	3.38	Castro Laboreiro	0.45	Lamas de Mouro	1.58										

Assessing the location and extent of HNVf_2_ required not only data on the intensity of the agricultural practices, but also on the structure and composition of the landscapes and the diversity of crops. Application and analysis of such indicators were carried out sequentially, with values for each parish ranked for the 12 parishes previously identified as farmlands (Table2). The diversity of such potential HNVf_2_ was analyzed first in relation to the Shannon's Evenness Index (SEI_p_; Table2). A threshold value of 0.60 for SEI_p_ excluded four parishes, Castro Laboreiro, Cubalhão, Lamas de Mouro, and Parada do Monte. The number of patches (NP_p_) and the mean shape index at the parish level (MSI_p_) were also analyzed to assess small-scale patterns in the landscape. Because these landscape metrics showed low variability, values for edge density (Ed_p_) were also analyzed (Landscape Elements set of indicators; Table2), and parishes exhibiting values under 300 ha were excluded as potential HNVf_2_. The exclusion of Cousso, Fiães, and Gave, after application of the ED_p,_ was further confirmed when considering the Crop Diversity indicators (Table2), as the aforementioned parishes were found to exhibit the lowest values of Shannon's Evenness Index for Crop Diversity (SDI_c_), even though Cousso and Gave exhibit the highest values for the number of crops (Scrop_p_).

Figure5 shows the spatially explicit representation of HNVf_1_ (a) and HNVf_2_ (b) areas. Overall, areas of HNVf_1_ appear to be distributed through the eastern part of the study area, whereas HNVf_2_ were found to be located mostly on the northwestern area. Table3 provides a comparison of the results from the minimum–maximum approach (Step 1) with the results from the further refinement using the proposed approach (i.e., including Steps 2 and 3). Whether the estimate of HNVf decreases, is maintained, or increases as a result of the refinements achieved from the proposed approach is also shown.

**Table 3:** Farmlands with high nature value for each Melgaço's parish according to the minimum–maximum approach (pHNVf_m_ and pHNVf_M_, respectively), and the HNVf_1_ and HNVf_2_ area identified following further refinement using the proposed spatially explicit approach. Comparison between the two approaches is expressed as trend for an increase (↑), decrease (↓), or no change (↔) in the calculation of HNV farmland areas. All areas are expressed in hectares (ha). n.a. stands for not applicable and refers to parishes where farmed areas are not dominant at the landscape level

Parish	pHNVf_m_ (ha)	pHNVf_M_ (ha)	HNVf_1_ (ha)		HNVf_2_ (ha)	
Alvaredo	4.17	216.02	–	**↓**	99.03	**↓**
Castro Laboreiro	4730.33	5242.61	4730.33	**↔**	–	**↓**
Chaviães	n.a.		n.a.			
Cousso	158.56	294.42	158.56	**↔**	–	**↓**
Cristóval	n.a.					
Cubalhão	673.32	798.81	673.32	**↔**	–	**↓**
Fiães	557.88	697.85	557.88	**↔**	–	**↓**
Gave	778.99	1006.15	778.99	**↔**	–	**↓**
Lamas de Mouro	1066.22	1176.45	1066.22	**↔**	–	**↓**
Paços	n.a.		n.a.			
Paderne	n.a.		n.a.			
Parada do Monte	903.10	1139.98	903.10	**↔**	–	**↓**
Penso	n.a.		n.a.			
Prado	–	116.06	–	**↓**	79.35	**↓**
Remoães	n.a.		n.a.			
Roussas	311.22	517.95	311.22	**↔**	162.26	**↓**
São Paio	298.55	533.07	298.55	**↔**	196.60	**↓**
Vila	–	87.10	–	**↔**	80.93	**↓**
Total (ha)	9525.66	11826.48	9478.18	↓	618.17	**↓**
Total HNVf (ha)	11826.48		10096.35			↓

Considering HNVf_1_, when comparing the two approaches, a decrease of area was observed in Alvaredo and Prado (cf. Figs.2 and 3), whereas in majority of parishes, the trend was for maintenance of the total area. Conversely, in the case of HNVf_2_, differences between the two approaches are expressed as a decrease for all targeted parishes. Overall, values for HNV farmlands, determined following the novel approach, resulted in a decrease of both HNVf_1_ and HNVf_2_ areas and a value of 1735.13 ha for the total HNVf extent.

### Species indicators and HNVf_3_ to support rare species

**Figure 6:**
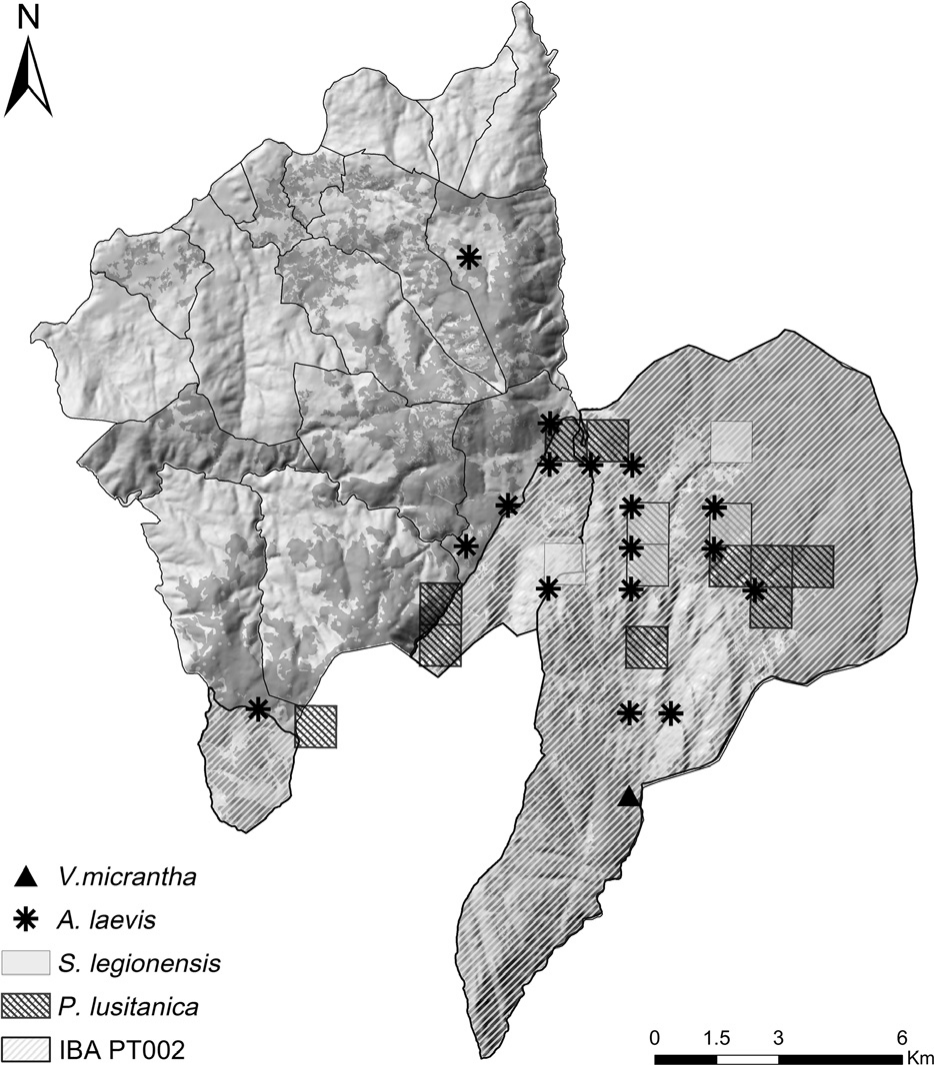
Representation of total HNVf (types 1 and 2) in relation to known occurrences of indicator plant species and Important Bird Areas (IBAs).

HNVf_3_ were assessed by applying a sensitivity test to the calculated HNVf areas, and the results are presented in Fig.6. In relation to the IBA PT002, located in the eastern part of the area, it comprises all of the extent of HNVf_1_ identified in Castro Laboreiro and parts of that in Lamas de Mouro and Gave. As for Indicator Species, the four squares of 1 km^2^ registered as occurrence areas for *Senecio legionensis* and 12 of 13 for *Paradisea lusitanica* were found to be partially within HNV farmlands type 1. *Veronica micrantha* occurrence in Castro Laboreiro was also found to be completely within HNVf type, whereas seven of 17 known populations of *Angelica laevis* were completely within targeted HNVf_1_ areas.

## Discussion

HNVf biodiversity hotspots constitute highly heterogeneous agriculture-dominated landscapes, containing a diversity of land cover and a widespread occurrence of semi-natural vegetation such as extensive grasslands (Bignal & McCracken 2000; Beaufoy 2008; Peppiette 2011; Weissteiner, Strobl & Sommer 2011). The HNV farmlands concept recognizes the positive relation between traditional farming systems and practices (traditionally low intensity and input) and habitats and species with high nature conservation value (Beaufoy et al. 1994; Oppermann et al. 2012; Ribeiro et al. 2014; Lomba et al. 2014).

HNV farmlands assessment in each EU Member State is mandatory under the Common Monitoring and Evaluation Framework (EC, 2005) and essential to evaluate the effectiveness of the EU and national Rural Development Programs (EC, 2005, EC 2006; Van Orshoven et al. 2012). However, the implementation and operationalization of such a complex concept have been hampered by a range of limitations (Andersen et al. 2003; EEA 2012), as the low spatial, temporal, and thematic resolution of the majority of data sources, for example, CORINE land cover (Paracchini et al. 2008; Doxa et al. 2010, 2012; Lomba et al. 2014), which potentially underestimate the specific features of local land use and biodiversity elements. The integration of both farming practices and landscape features related data, and the implementation of cross-validation procedures in relation to biodiversity indicators (Bailey et al. 2007; Doxa et al. 2012) has been highlighted as major challenges to be considered within national and/or regional assessments (EENRD 2009).

To address such challenges, and in agreement with the bottom-up approach proposed by Lomba et al. (2014), we implemented a spatially explicit framework to assess the extent and location of farmlands with high nature value and hence the definition of priority areas for maintenance of agro-biodiversity in the European countryside. In contrast to other approaches for HNV farmlands assessment (Peppiette 2011; Oppermann et al. 2012; Lomba et al. 2014), the framework allowed both the identification of HNVf at the level of the local administrative unit, that is, Melgaço municipality, and the identification of the extent of each individual HNVf types (Fig.5; after Andersen et al. 2003). In particular, the framework enabled the identification of LAUs where farmlands are dominant in the landscape, which resulted in a decrease of HNV farmlands extent (types 1 and 2), when compared to approaches previously proposed (Paracchini et al. 2008; Lomba et al. 2014). Moreover, it also enabled the validation of the calculated extent of HNVf using species whose survival relies on extensively managed farmlands (HNVf_3_; cf. Fig.6).

The advantages of the envisioned framework over other methods (see Lomba et al. 2014 for a comprehensive review of different methods) are the result of considering spatially explicit indicators informing not only on distinct biodiversity levels, but also on landscape structure, composition and diversity, and the intensity and diversity of crops and associated practices. Using data with the best spatial and temporal resolution available for each LAU, we ensure that the most detailed indicators were applied to map the extent of HNV farmlands in any targeted area. The proposed sets of indicators followed the recommendations of EU agro-environmental indicators (Paracchini et al. 2006), while also relying on data sources that are periodically updated, for example, detailed land cover map (Associação de Municípios do Vale do Minho 2009) and agrarian statistics (INE 2009). As a result, our approach also ensures that the extent and dynamics of HNV farmlands can also be monitored over time, thus meeting RDP program requirements (EC 2006). However, results will be at large extent a trade-off between the thematic, spatial, and temporal resolution of the datasets available in each LAU, region and ultimately Member State. Even so, by mobilizing the best data available to inform on the proposed indicators (within a collaborative network for data exchanging; Lomba et al. 2014), it is assured that the best HNVf assessment is achieved for each time period.

Our approach allows the identification of areas relevant for the conservation, maintenance, and eventually enhancement of agro-biodiversity. Melgaço is currently under the designation of EU Less Favoured Areas (LFA) is a EU legal designation, that is, an area where agriculture is constrained by natural handicaps, and our results highlight the decreasing gradient of natural value from the eastern LAUs, for example, Castro Laboreiro (Beilin et al. 2014) to western LAU, thus the first highlighted as essential for both conservation of habitats (expressed as a high proportion of HNVf_1_; Fig.5A) and species (both birds and plants; Fig.6). Conversely, areas of HNVf_2_ appear as complementary areas in northwestern LAUs, near the more urbanized areas (cf. Fig.5B). Such outcomes constitute a step forward toward a more precise targeting of landscapes for agri-environment schemes, as they allow a more accurate discrimination of areas within landscapes that have a higher value for nature conservation. In fact, such discrimination is built not only on the ecological value of the farmlands but also on the extensive and/or traditional character of the agricultural practices.

An added value of the approach is therefore a more refined identification of areas where land-sharing for biodiversity conservation and/ or enhancement in the European countryside may be relevant or even essential and is not expected to cause conflicts with other (more intensive) land uses (Egan & Mortensen 2012; Navarro & Pereira 2012). Such refinement can be useful to define priority areas to be targeted in rural landscapes where farmers can benefit from agri-environmental payments, to support the identification of areas with potential to maintain agricultural-dependent habitats, and ultimately to contribute to more effective RDPs. In the specific case of Melgaço, which is fully under the status of Less Favoured Areas (LFA) is a EU legal designation, highlighting areas with higher natural value and targeting them under agri-environmental payments may halt the agricultural abandonment trend in the area, which will be essential if we aim to maintain such areas and their agro-biodiversity in the future.

While at the local and regional level, the informed targeting of rural landscapes can enhance the ability of territories to support agro-biodiversity maintenance and other ecosystem services (including provisioning, regulating, and cultural), and to support an informed targeting of rural landscapes to be supported by agro-environmental payments, our approach can be applied across the EU countryside, thus contributing to a more realistic mapping and assessment of HNVf at the EU level.

Even if the results of our approach are promising, there is room for improvement. The application of the spatially explicit framework to other farmlands, where the socio-ecological context is distinct, will allow testing of not only the sensitivity, but also of the transferability and simplicity, of the proposed sets of indicators. In addition, the detail of some of the options used in our approach will need to be altered to reflect variation across European farmlands, for example, the accepted threshold for the intensity of agricultural practices (Oppermann et al. 2012), and /or the targeted classes of land cover that reflect natural and semi-natural agricultural habitats (Paracchini et al. 2006, 2008; EEA 2012). Nevertheless, the application of a common approach will mean that the extent and distribution of different HNVf types will be more easily compared and contrasted at an EU level (Lomba et al. 2014). In addition, there is also scope to test the framework under scenarios of land use change (e.g., Verburg & Overmars 2009), to assess its ability to detect changes both in the condition and dynamics of HNVf, and thus to anticipate the loss of important areas for agro-biodiversity maintenance.

This proposed framework is, to our knowledge, one of only few that focus on the spatially explicit identification of the different types of HNV farmlands, thus complying with the EU need for strategic monitoring of the EU countryside. We advocate that the implementation of this framework should be linked strongly to a collaborative European network (Lomba et al. 2014) that can promote the integration and exchange of data from different sources and across scales. The development of such an approach is essential if the range of threats facing HNVf landscapes is to be identified and monitored properly from local to European level. Moreover, this would then allow relevant agri-environment measures to be developed and implemented at the scale required to help maintain the habitats and species of high nature conservation value that are intimately associated with those landscapes.

## Supporting Information

**Appendix S1.** Utilised Agricultural Area (UAA) spatially explicit definition in relation to land cover classes.

**Appendix S2.** Spatially explicit definition of Areas of Natural Constrains for Agriculture.

**Appendix S3.** Indicators used to implement the spatially explicit approach to assess the extent of High Nature Value farmlands.

**Appendix S4.** Rank of targeted parishes according to shares of farmland versus forests areas and extensive practices indicators within Melgaço Municipality.
