# Supplementary material for: Reconciling nature conservation and traditional farming practices: a spatially explicit framework to assess the extent of High Nature Value farmlands in the European countryside
Source: Ecol Evol. 2015 Feb 5;5(5):1031–44. doi: 10.1002/ece3.1415 (PMC4364818; doi:10.1002/ece3.1415)
Supplement: Supplementary file 1 [file ece30005-1031-sd1.doc]

**Appendix S1. Utilised Agricultural Area (UAA) spatially-explicit definition in relation to land cover classes and High Nature Value Farmlands potential.**

The highest spatial and thematic resolution available land-cover map for the study area (COS2009; Associação de Municípios do Vale do Minho 2009) was considered and used to build a spatially-explicit representation of farmland areas with potential to be High Nature Value (High Nature Value farmlands, hereafter HNVf), following the European Commission recommendations (Paracchini *et al.* 2008; EENRD 2009). Initially, classes potentially corresponding to farmlands were identified as Utilised Agricultural Area (area used for farming, including arable land, permanent grassland, permanent crops and other agricultural land such as kitchen gardens, UAA; EENRD 2009), and the remaining ones ascertained as *not farmlands* *(n.f.; including* all land cover classes not eligible as farmed areas e.g. artificial surfaces and forests).

Such discrimination is presented in Table S1 to assure replicability. Afterwards, classes with potential to express farmlands (UAA in Table S1) and other classes of semi-natural vegetation were identified and their potential to reflect HNV and non HNVf disentangled e.g. Paracchini *et al.* 2008; Oppermann, Beaufoy & Jones 2012. Such discrimination was built on the assumption that some predominant land-cover types are characteristic of each category of HNV farmlands, e.g. in the case of semi-natural vegetation, types of cropped land, and their typical spatial coverage and distribution at the landscape level e.g. see Paracchini *et al.* 2008. As the available land cover map do not convey information regarding land use intensity, a *Minimum-Maximum* selection approach (as described previously by Andersen *et al.* 2003) was implemented so that an preliminary analysis of putative “extremes” within which HNVf was likely to occur could be defined. Overall, the *Minimum selection* includes only the classes of land cover which are made up primarily of HNV land, while the *Maximum* selection included all classes with some farmed HNV land. As so, the *Maximum* selection is expected to contain much non-HNV land, whilst the *Minimum* inevitably would exclude some probable HNV land.

***Table S1*** - Land Cover classes present in the study area, correspondence to broad land-cover classes and probable approach to land use, and their likelihood to correspond to High Nature Value farmlands (HNVf). Presented categories are those used for Corine Land Cover, and Classes are the applied for mapping COS 2009; (Associação de Municípios do Vale do Minho 2009)). *Minimum HNVf* areas comprise areas with very high likelihood of being farmlands with high conservation value (thus, comprising land cover classes which are made up primarily of HNVf landscapes), whereas the *Maximum HNVf* coincide with those farmed areas where some maybe associated with HNVf (and thus comprise land cover classes in which farmed areas are not the predominant land use). *n.f.* stands for *not farmlands* and includes all other land cover classes that are not eligible as HNVf, as they do not include potentially farmed areas. (Beaufoy 2008; Paracchini *et al.* 2008). Classes of land cover that can be grazeable (this is, fodder areas) are also highlighted as even if they are only partially coincident with the Utilised Agricultural Area (UAA), they are relevant for the estimation of the Livestock density index (LSD).

| **Land Cover classes** | | **Description of land-cover classes in relation to land-use** | ***n.f.*** | **farmlands** | **UAA** | **Grazeable** | ***non* HNVf** | **HNV farmlands** | |
| --- | --- | --- | --- | --- | --- | --- | --- | --- | --- |
|  |  |  |  |  |  |  |  | ***Minimum* HNVf** | ***Maximum* HNVf** |
| Artificial surfaces | | Urban fabric | x |  |  |  |  |  |  |
|  |  | Infrastructures and equipments | x |  |  |  |  |  |  |
|  |  | Mine, dump, construction sites and other degraded areas | x |  |  |  |  |  |  |
| Agricultural Areas | Arable land | Non-irrigated arable land, permanently irrigated land, rice fields and others |  | x | x | x | x |  |  |
|  |  | Mosaics of arable land and grasslands |  | x | x | x |  | x | x |
|  | Permanent crops | Vineyards |  | x | x |  | x |  |  |
|  |  | Vineyards and arable land |  | x | x | x | x |  |  |
|  |  | Vineyards and orchards |  | x | x | x | x |  |  |
|  |  | Orchards |  | x | x | x |  |  | x |
|  |  | Fruit trees + Olive groves |  | x | x | x |  |  | x |
|  | Pasture | Grasslands |  | x | x | x |  | x | x |
|  | Heterogeneous agricultural areas | Annual crops + Vineyards |  | x | x |  |  |  | x |
|  |  | Annual crops + Fruit trees |  | x | x |  |  |  | x |
|  |  | Complex crop mosaics |  | x | x |  |  |  | x |
|  |  | Agro-forestry areas with broadleaved trees |  | x | x | x |  |  | x |
|  |  | Other agro-forestry areas with planted trees |  | x | x | x |  |  | x |
| Forest and semi-natural areas | Forests | Broad-leaved forests | x |  |  |  |  |  |  |
|  |  | Coniferous forests | x |  |  |  |  |  |  |
|  |  | Mixed forests | x |  |  |  |  |  |  |
|  | Scrub and/or herbaceous vegetation associations | Heathlands located in areas under low or no natural constraints for agriculture | x | x |  | x |  | x | x |
|  |  | Other heathlands and transitional woodland-shrub | x |  |  |  |  |  |  |
|  |  | Degraded forests | x |  |  | x |  |  |  |
|  | Open spaces with little or no vegetation | Sparsely vegetated | x |  |  | x |  |  |  |
| Water bodies | | Water courses | x |  |  |  |  |  |  |

**References**

Andersen, E., Baldock, D., Bennett, H., Beaufoy, G., Bignal, E., Bouwer, F., Elbersen, B., Eiden, G., Giodeschalk, F., Jones, G., McCracken, D., Nieuwenhuizen, W., Eupen, M.v., Hennekes, S. & Zervas, G. (2003) Developing a high nature value farming area indicator : final report. pp. 75.

Associação de Municípios do Vale do Minho (2009) Promoção e Sustentabilidade das Paisagens do Vale do Minho (GAEPC/ON.2/2008-2011).

Beaufoy, G. (2008) HNV Farming – Explaning the concept and interpreting EU and National Policy Commitments. *European Forum on Nature Conservation and Pastoralism*, pp. 15.

EENRD (2009) Guidance Document-The Application of the High Nature Value Impact Indicator Programming Period 2007-2013. (ed. E. Communities), pp. 45. European Evaluation Network for Rural Development, Brussels.

Oppermann, R., Beaufoy, G. & Jones, G. (2012) *(eds). High Nature Value farming in Europe. 35 European countries: experiences and perspectives. Verlag Regionalkultur, Ubstadt-Weiher, Germany.*

Paracchini, M.L., Petersen, J.-E., Hoogeveen, Y., Bamps, C., Burfield, I. & Van Swaay, C. (2008) High Nature Value Farmland in Europe - An estimate of the distribution patterns on the basis of land cover and biodiversity data. JRC Scientific and Technical Reports (ed. O.f.O.P.o.t.E. Communitites), pp. 87pp. Joint Research Centre - Institute for Environment and Sustainability, Luxembourg.
