# Supplementary material for: Reconciling nature conservation and traditional farming practices: a spatially explicit framework to assess the extent of High Nature Value farmlands in the European countryside
Source: Ecol Evol. 2015 Feb 5;5(5):1031–44. doi: 10.1002/ece3.1415 (PMC4364818; doi:10.1002/ece3.1415)
Supplement: Supplementary file 2 [file ece30005-1031-sd2.doc]

**Supporting Information S2.** Spatially-explicit definition of Areas of Natural Contraints for Agriculture

**Areas of Natural Constraints for agriculture** (ANC) were derived in compliance with the new approach adopted by the European Commission (hereafter EC). Overall, the new proposed framework to target the aid to farmers in areas with natural handicaps (Eliasson et al., 2007; European Commission, 2009), is built on three sets of common biophysical criteria, including soil, climate and terrain, and aims to a more objective and clear classification of such areas. From the proposed sets of criteria, available information from soil and terrain was selected to achieve a map expressing classes of natural constraint in the study area, and are described in Table S2.

**Table *S2*.** Criterions applied to map Areas of Natural Constrains (ANC), with allusion to the rationale under each of the criteria selected, thresholds considered, source and resolution of the spatial information used and supporting bibliographic references. cm, centimeters m, meters; %, percentage.

| **Set of criteria** | **Criterion** | **Definition** | **Threshold** | **Source/ spatial resolution** | **Reference** |
| --- | --- | --- | --- | --- | --- |
| **Soil** | Drainage | Areas which are water logged for significant duration of the year, expressing poor soil drainage (lack of gaseous oxygen in soil for root growth or land not accessible for tillage). | *n.a.*  (classes d3 and d4; Agroconsultores, 1999) | Carta de Solos e Aptidão da terra para a agricultura em Entre-Douro-e-Minho (1:25000) | Agroconsultores, 1999; Eliasson et al., 2007 |
|  | Rooting depth | Land areas which have depth limitations (expressed as cm) of the surface caused by the presence of coherent, hard rock or hard-pans. | <30cm  classe 4; Agroconsultores, 1999 | Carta de Solos e Aptidão da terra para a agricultura em Entre-Douro-e-Minho (1:25000) | Agroconsultores, 1999; Eliasson et al., 2007 |
| **Soil and Climate** | Soil moisture | Soil hydric deficit, expressing the available water in soil, related to climatic and soil inherent properties. | classes 3 and 4; Agroconsultores, 1999 | Carta de Solos e Aptidão da terra para a agricultura em Entre-Douro-e-Minho (1:25000) | Agroconsultores, 1999; Eliasson et al., 2007 |
| **Terrain** | Slope (%) | Percentage of elevation with respect to planimetric distance. | >15% | Digital Elevation Model (pixel size: 20m) | Projecto SIGNII  http://sitgaideg.xunta.es/sign/ |

Specifically, spatially-explicit information on each of the targeted criterion (soil, climate and terrain related; see Table S2) was converted to raster files (spatial resolution determined using as rule of thumb the lowest resolution of all data available) to allow more adequate calculations. Due to the extent of the study area (< 50 x 50 km of pixel; Eliasson et al., 2007), climate related criterion were not considered for this exercise, as climatic conditions were considered to be rather homogeneous. Each raster, consisting on several classes according to the source data (Agroconsultores, 1999), was reclassified in classes according to the thresholds defined by Eliasson et al., 2007 (Figure S2a,b,c,d). We assumed that below the threshold, low or moderate natural constraints occur and attributed to the corresponding classes (see Table S2) the value “0”. In the case of classes considered against the threshold established, it was considered that they would correspond to areas with high or severe natural constraints and thus they were reclassified with a “1” value. To achieve an ANC map, the individual raster’s for each criterion considered were summed, and two classes expressing natural areas from low constraints to areas with severe natural constraints obtained, with the last reflecting where the highest number of natural constraints occur simultaneously (see Figure S2e).

**Figure S2**. Information on soil (drainage and rooting depth), soil and climate (soil moisture) and terrain (slope) data criterion, applied to achieve a map expressing Areas of Natural Constrains (ANC) in the study area.

b)

e)


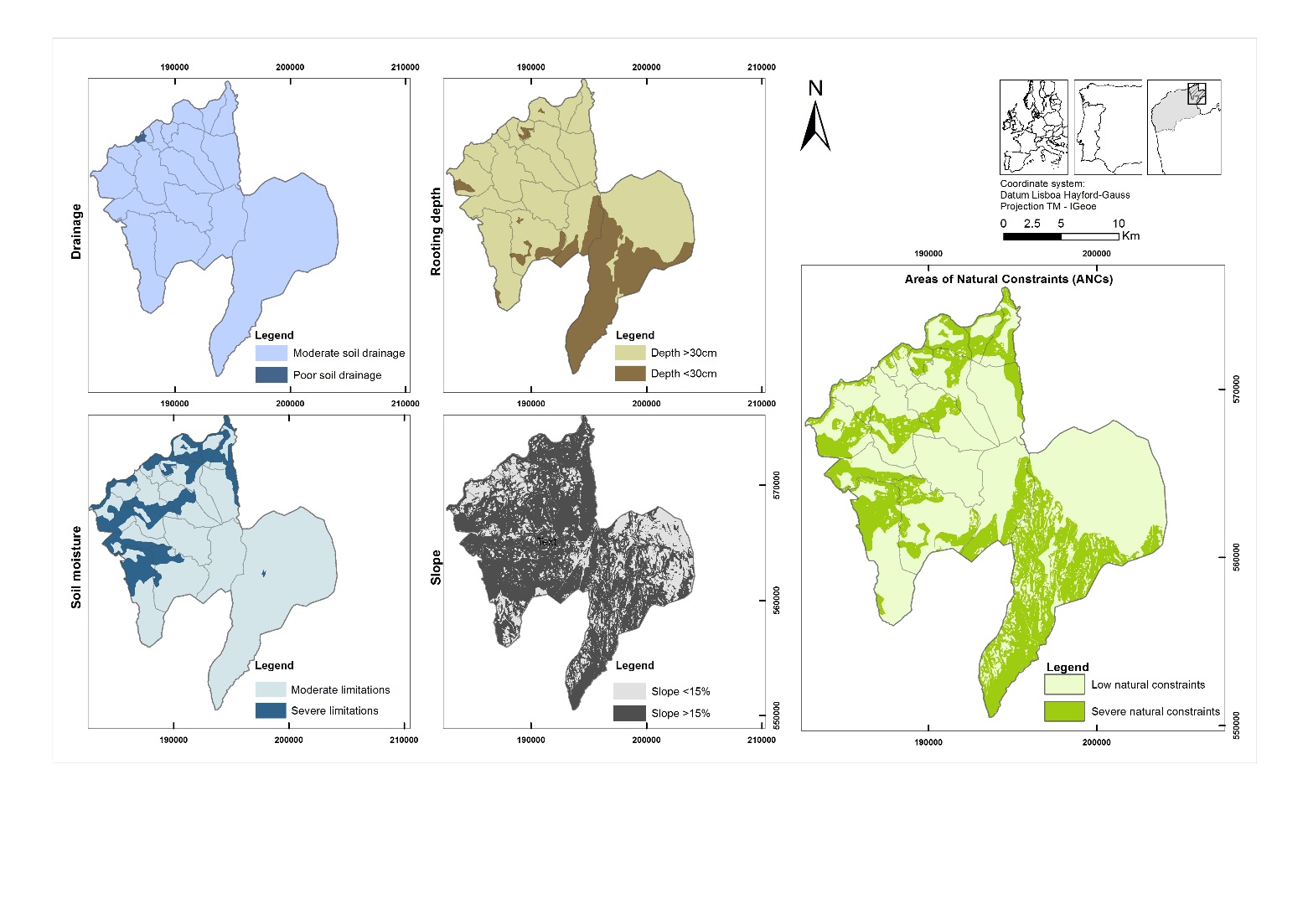


a)

c)

d)

**References**

Agroconsultores, C.e.G., 1999. Carta dos solos e carta de aptidão da terra de entre-douro e Minho, in: DRAEDM (Ed.).

Eliasson, Å., Terres, J.-M., Bamps, C., 2007. (eds) (2007). Common Biophysical Criteria for Defining Areas which are Less Favourable for Agriculture in Europe. Proceedings from the Expert Meeting 19-20th of April, 2007. The Institute for Environment and Sustainability Joint Research Centre, Ispra (Italy).

European Commission, 2009. Communication from the Commission: "Towards a better targeting of the aid to farmers in areas with natural handicaps". Brussels, COM(2009) 161 final. 11pp.
