# Supplementary material for: Reconciling nature conservation and traditional farming practices: a spatially explicit framework to assess the extent of High Nature Value farmlands in the European countryside
Source: Ecol Evol. 2015 Feb 5;5(5):1031–44. doi: 10.1002/ece3.1415 (PMC4364818; doi:10.1002/ece3.1415)
Supplement: Supplementary file 3 [file ece30005-1031-sd3.docx]

**Supporting Information S3.** Indicators used to implement the spatially-explicit approach to assess the extent of High Nature Value farmlands

**Table S3.** Indicators used to implement the spatially-explicit approach to assess the extent of High Nature Value farmlands. Indicators expressing landscape characteristics (Landscape elements), the intensity (Extensive practices) and the diversity (Crop diversity) of farming practices, as well as occurrence of Indicator Species, are described and the rationale underlying their selection presented. %, stands for the percentage; *n.a.*, non-applicable.

| **Designation** | **Code(s)** | **Description** | **Units** | **Source and Resolution** | **Rationale** | **HNVf type** | **Reference** |
| --- | --- | --- | --- | --- | --- | --- | --- |
| **Landscape Elements (LE)** |  |  |  |  |  |  |  |
| Natural constraints for agriculture | ANC_p_ | Areas with natural constraints to agricultural per parish; see Table and Figure in appendix S2 for detailed information) | *n.a.* | *n.a.* | Location of areas with natural constraints enable to determine more suitable areas for agriculture (UAA), and zonation of areas with potential to express HNVf. | HNVf type 1  HNVf type 2 | (Van Orshoven, Terres & Tóth 2012) |
| Farmlands dominance in the landscape | P.UAA_p_  P.Forest_p_ | To define the dominance of farmlands at the level of the parish landscape, the percentage of area covered by UAA (P.UAA_p_) was analysed against the area covered by forests (P.Forest_p_) | % | Computed based on the land-cover map (Associação de Municípios do Vale do Minho 2009) in ArcMap 10.2 (ESRI 2009-2013) | Farmed areas where the percentage cover of UAA was similar or lower than those occupied by forests, were not considered as agricultural-dominated landscapes. | HNVf type 1  HNVf type 2  HNVf type 3 | (Beaufoy, Baldock & Clarke 1994; Andersen *et al.* 2003) |
| Minimum-Maximum HNV farmland areas | pHNVf_m_ | Farmlands more likely to represent HNVf (Minimum, pHNVf_m_) in the study area (Melgaço municipality) and *per* parish (derived from the land-cover map; see Table and Figure in appendix S1 for supporting information) | *n.a.* | *n.a.* | Detailed land-cover information allows the establishment of spatially-explicit UAA and potential HNVf maps, by identifying land-cover classes likely to correspond to farmlands. | HNVf type 1 | (Andersen *et al.* 2003; Paracchini *et al.* 2006) |
|  | pHNVf_M_ | Farmlands with moderate potential to represent HNVf (Maximum, pHNVf_M_) in the study area (Melgaço municipality) and *per* parish (derived from the land-cover map; see Table and Figure in appendix S1 for supporting information) |  |  |  | HNVf type 2 |  |
| Shannon’s Diversity Index | SDI_p_ | Expresses information about area composition, considering both the number of types of land cover present and their relative proportion in the parish landscape. | *n.a.* | Obtained by analysis of the land-cover map (Associação de Municípios do Vale do Minho 2009) through implementation of Patch Analyst (Rempel, Kaukinen. D. & Carr. 2012) for ArcMap 10.2 (ESRI 2009-2013). | When the landscape is diverse and the area of the different land cover types is evenly distributed the landscape will have a higher diversity value than landscapes where only one or two types dominate. | HNVf type 2 | (Tscharntke *et al.* 2005; BioBio 2012) |
| Shannon Evenness Index | SEI_p_ | Shannon Evenness index expresses the landscape diversity at the parish level. Obtained dividing the Shannon diversity index by its maximum varies between 0 and 1. |  |  |  |  |  |
| Patch number | NP_p_ | Total number of patches of all land cover types present at the parish landscape level. |  |  | The number of patches is often used as an indicator for landscape fragmentation, often highlighted as beneficial for biodiversity in agricultural landscapes. |  | (Aavik & Liira 2009; Armengot *et al.* 2011) |
| Mean Shape Index | MSI_p_ | Expresses the complexity of patch shape considering all land cover types in the parish landscape. Lower values (near 1) depict more regular shapes, whereas higher values refer to shapes more close to nature. |  |  | Higher MSI values occur in natural and semi-natural landscapes. |  | (Tscharntke *et al.* 2005) |
| Edge density | ED_p_ | Measures the amount of edges in relation to the landscape area (parish). | m/ha (meters per hectare) |  | Linear habitats, as e.g. edges and hedgerows, are considered to be semi-natural as they have been related to be relevant to wildlife maintenance. |  | (BioBio 2012) |
| **Extensive Practices (EP)** |  |  |  |  |  |  |  |
| Livestock Density Index | LSI_p_ | Expresses the number of livestock units (LSU; cattle, sheep, goats and *equidae*, as defined according to EUROSTAT per hectare of the UAA determined per parish. | LSU_i_/ha of UAA (livestock density per hectare of UAA) | (INE 2009) | Used as a proxy for agricultural intensification. Lower values of LSU_i_ highlight the dominance of semi-natural forage, including grasslands and often, scrub, woodlands, or a combination of several types. | HNVf type 1  HNVf type 2 | (Pointereau *et al.* 2007; EENRD; BioBio) |
| Share of Irrigated Area | Irrig_p_ | Determined as the share of irrigated area per total of UAA in each parish. | % |  | Used as proxy for agricultural intensification. Parishes with a high share of irrigated area are assumed to be under more intensive agricultural practices. |  | (Pointereau *et al.* 2010) |
| **Crop Diversity (CD)** |  |  |  |  |  |  |  |
| Shannon Evenness Index for Crop Diversity | SEI_c_ | Measures simultaneously changes in crop diversity (those included as arable land, permanent crops and permanent grasslands) and evenness in their distribution at the parish landscape level. Its value varies between 0 (no diversity; i.e. a single crop type), and 1 (maximum crop diversity combined with complete evenness). | *n.a.* | (INE 2009) | Crop diversity, associated with low inputs and a network of natural and/or semi-natural features express a high nature value for biodiversity conservation in farmlands. Crop diversity, expressed as richer crop composition and the more equal the shares (maximum value, 1), is considered to enhance biodiversity in farmlands. | HNVf type 2 | (Andersen *et al.* 2003; Paracchini *et al.* 2008; BioBio 2012) |
| Crop Richness | SCrop_p_ | Number of distinct types of crops cultivated as arable lands, permanent crops and permanent grasslands, per parish. | Number of crops/parish |  | Lower specialisation of the cropping patterns within agricultural landscapes can contribute to higher levels of agro-biodiversity. | HNVf type 2 | (BioBio 2012) |
| **Indicator Species (Isp)** |  |  |  |  |  |  |  |
| Important Bird and Biodiversity Areas | IBAs | IBAs are the sites needed to ensure the survival of viable populations of most of the world’s bird species. | *n.a.* | 1:20 000  (SPEA 2003) | The National Park (Serras da Peneda Gerês; PT002) is an IBA under the criteria B2 and C6 for bird species. Further, this area holds a representative proportion of other levels of biodiversity (e.g. natural and semi-natural habitats) | HNVf type 3 | (Andersen *et al.* 2003; Paracchini *et al.* 2008) |
| Indicator Plant Species | IPs | Plant species under conservation status (Habitats Directive) depending on extensive farmlands maintenance. | *n.a.* | CIBIO database | Plant species endemic from the Iberian Peninsula (*Angelica laevis* J. Gay, *Paradisea lusitanica* (Cout.) Samp, *Senecio doria* L. subsp. *Legionensis* (Lange), and *Veronica micrantha* Hoffmanns. & Link), known to depend on farmlands, can provide additional information for the identification of HNVf areas. |  |  |

**References**

Aavik, T. & Liira, J. (2009) Agrotolerant and high nature-value species—Plant biodiversity indicator groups in agroecosystems. *Ecological Indicators,* **9,** 892-901.

Andersen, E., Baldock, D., Bennett, H., Beaufoy, G., Bignal, E., Bouwer, F., Elbersen, B., Eiden, G., Giodeschalk, F., Jones, G., McCracken, D., Nieuwenhuizen, W., Eupen, M.v., Hennekes, S. & Zervas, G. (2003) Developing a high nature value farming area indicator : final report. pp. 75.

Armengot, L., José-María, L., Blanco-Moreno, J.M., Romero-Puente, A. & Sans, F.X. (2011) Landscape and land-use effects on weed flora in Mediterranean cereal fields. *Agriculture, Ecosystems & Environment,* **142,** 311-317.

Associação de Municípios do Vale do Minho (2009) Promoção e Sustentabilidade das Paisagens do Vale do Minho (GAEPC/ON.2/2008-2011).

Beaufoy, G., Baldock, D. & Clarke, J. (1994) *The Nature of Farming - Low Intensity Farming Systems in Nine European Countries*. IEEP, London.

BioBio (2012) Biodiversity indicators for organic and low-input farming systems” (KBBE 227661). (eds Felix Herzog, Katalin Balázs, Peter Dennis, Ilse Geijzendorffer, Jürgen K. Friedel, Philippe Jeanneret, Max Kainz & P. Pointereau).

EENRD (2009) Guidance Document-The Application of the High Nature Value Impact Indicator Programming Period 2007-2013. (ed. E. Communities), pp. 45. European Evaluation Network for Rural Development, Brussels.

ESRI (2009-2013) ArcMap 10.2. Environmental Systems Research Institute Inc.

Paracchini, M.L., Petersen, J.-E., Hoogeveen, Y., Bamps, C., Burfield, I. & Van Swaay, C. (2008) High Nature Value Farmland in Europe - An estimate of the distribution patterns on the basis of land cover and biodiversity data. *JRC Scientific and Technical Reports* (ed. O.f.O.P.o.t.E. Communitites), pp. 87pp. Joint Research Centre - Institute for Environment and Sustainability, Luxembourg.

Paracchini, M.L., Terres, J.-M., Petersen, J.-E. & Hoogeveen, Y. (2006) Background Document on the Methodology for Mapping High Nature Value Farmland in EU27. *European Commission Directorate General Joint Research Centre and the European Environment Agency*, pp. 32.

Pointereau, P., Doxa, A., Coulon, F., Jiguet, F. & Paracchini, M.L. (2010) High Nature Value Farmland and Common Bird Indicators. Spatial and Temporal Modifications in Agricultural Practices and Bird Communities. Luxembourg: Office for Official Publications of the European Communities.

Pointereau, P., Paracchini, M.L., Terres, J.-M., Jiguet, F., Bas, Y. & Biala, K. (2007) Identification of High Nature Value Farmland in France through Statistical Information and Farm Practices Surveys. Luxembourg: Office for Official Publications of the European Communities. *EUR – Scientific and Technical Research series*, pp. 65.

Rempel, R.S., Kaukinen. D. & Carr., A.P. (2012) Patch Analyst and Patch Grid. Ontario Ministry of Natural Resources. Centre for Northern Forest Ecosystem Research, Thunder Bay, Ontario.

Tscharntke, T., Klein, A.M., Kruess, A., Steffan-Dewenter, I. & Thies, C. (2005) Landscape perspectives on agricultural intensification and biodiversity - ecosystem service management. *Ecology Letters,* **8,** 857-874.

Van Orshoven, J., Terres, J.-M. & Tóth, T.e. (2012) Updated common bio-physical criteria to define natural constraints for agriculture in Europe. Definition and scientific justification for the common biophysical criteria; Technical Factsheets. JRC Scientific and Technical reports. ISBN 978-92-79-23066-0.
