# Supplementary material for: Reconciling nature conservation and traditional farming practices: a spatially explicit framework to assess the extent of High Nature Value farmlands in the European countryside
Source: Ecol Evol. 2015 Feb 5;5(5):1031–44. doi: 10.1002/ece3.1415 (PMC4364818; doi:10.1002/ece3.1415)
Supplement: Supplementary file 4 [file ece30005-1031-sd4.docx]

**Supporting Information S4.** Rank of targeted parishes according to shares of farmland *versus* forests areas and extensive practices indicators within Melgaço Municipality.

Spatially-explicit expression of the share of farmed (P.UAA_p_) and forested (P.Forest_p_) areas in each of the 18 Melgaço’s parishes.

**Table S4.1.** Rank of the relationship between shares of farmland (P.UAA_p_) *versus* forests (P.Forest._p_) areas for each parish within Melgaço Municipality. Areas are expressed as hectares (ha). Share of farmlands (P.UAA_p_) and forests (P.Forest_p_) are presented as percentage (%) of the respective cover in relation to the parish area (T_area_ ). *n.f.* stands for not farmland areas. Grey area in the table establishes the threshold considered.

| **Parish** | **T_area_ (ha)** | **n.f. (ha)** | **UAA_p_ (ha)** | **Forest_p_ (ha)** | **P.UAA_p_ (%)** | **P.Forest_p_ (%)** |
| --- | --- | --- | --- | --- | --- | --- |
| Cubalhão | 1161.79 | 362.98 | 798.81 | 86.00 | 68.76 | 7.40 |
| Lamas de Mouro | 1764.02 | 587.57 | 1176.45 | 76.69 | 66.69 | 4.35 |
| Parada do Monte | 1822.15 | 682.17 | 1139.98 | 296.22 | 62.56 | 16.26 |
| Fiães | 1121.30 | 423.45 | 697.85 | 194.59 | 62.24 | 17.35 |
| Castro Laboreiro | 8845.12 | 3602.51 | 5242.61 | 769.71 | 59.27 | 8.70 |
| Gave | 1863.98 | 857.83 | 1006.15 | 502.39 | 53.98 | 26.95 |
| Roussas | 965.21 | 447.26 | 517.95 | 169.66 | 53.66 | 17.58 |
| São Paio | 995.20 | 462.13 | 533.07 | 184.18 | 53.56 | 18.51 |
| Alvaredo | 436.43 | 220.41 | 216.02 | 129.84 | 49.50 | 29.75 |
| Vila | 185.69 | 98.59 | 87.10 | 30.39 | 46.91 | 16.37 |
| Prado | 262.37 | 146.31 | 116.06 | 75.23 | 44.23 | 28.67 |
| Cousso | 723.50 | 429.08 | 294.42 | 175.26 | 40.69 | 24.22 |
| Paderne | 1285.05 | 783.18 | 501.87 | 495.20 | 39.05 | 38.54 |
| Remoães | 104.40 | 64.83 | 39.57 | 40.57 | 37.91 | 38.86 |
| Cristóval | 555.71 | 353.51 | 202.20 | 181.69 | 36.39 | 32.70 |
| Paços | 367.47 | 259.93 | 107.54 | 138.53 | 29.26 | 37.70 |
| Penso | 885.42 | 637.82 | 247.60 | 239.34 | 27.96 | 27.03 |
| Chaviães | 480.45 | 355.71 | 124.74 | 182.78 | 25.96 | 38.04 |

Table S4.2 contains the values established as thresholds for the indicators of intensity of agricultural practices. Livestock density and the share of irrigated areas at the parish level were analysed to assess High Nature Value farmlands type 1. As all values for livestock density index (LSI_p_) were found to be under 0.2LSU/ha, values for the share of irrigated area (Irrig_p_ ) above 15% of the total UAA were considered as a threshold for assessing HNVf_1_. As a result Vila, Prado and Alvaredo were excluded as areas legible for HNVf type 1.

**Table S4.2.** Rank of parishes according to extensive practices indicators, livestock density index (LSI_p_), and share of irrigated area (Irrig_p_). Grey area in the table establishes the threshold considered to assess HNVf type 1 extent.

| **Parish** | **Extensive Practices Set of Indicators** | | |
| --- | --- | --- | --- |
|  | **LSI_p_ (LSU/ha)** | **Parish** | **Irrig_p_ (%)** |
| Lamas de Mouro | 0.03 | Lamas de Mouro | 2.38 |
| Roussas | 0.10 | Castro Laboreiro | 2.50 |
| Alvaredo | 0.11 | Cubalhão | 3.38 |
| São Paio | 0.11 | Fiães | 5.45 |
| Remoães | 0.12 | Parada do Monte | 6.14 |
| Cousso | 0.14 | Gave | 6.16 |
| Gave | 0.14 | Cousso | 10.19 |
| Prado | 0.14 | São Paio | 11.44 |
| Castro Laboreiro | 0.16 | Roussas | 12.94 |
| Fiães | 0.19 | Vila | 34.44 |
| Parada do Monte | 0.19 | Prado | 40.50 |
| Vila | 0.19 | Alvaredo | 53.24 |
